# Spin-dependent Seebeck Effect, Thermal Colossal Magnetoresistance and Negative Differential Thermoelectric Resistance in Zigzag Silicene Nanoribbon Heterojunciton

**DOI:** 10.1038/srep10547

**Published:** 2015-05-22

**Authors:** Hua-Hua Fu, Dan-Dan Wu, Zu-Quan Zhang, Lei Gu

**Affiliations:** 1 College of Physics, Huazhong University of Science and Technology, Wuhan 430074, People’s Republic of China; 2 Wuhan National High Magnetic field center, Huazhong University of Science and Technology, Wuhan 430074, People’s Republic of China

## Abstract

Spin-dependent Seebeck effect (SDSE) is one of hot topics in spin caloritronics, which examine the relationships between spin and heat transport in materials. Meanwhile, it is still a huge challenge to obtain thermally induced spin current nearly without thermal electron current. Here, we construct a hydrogen-terminated zigzag silicene nanoribbon heterojunction, and find that by applying a temperature difference between the source and the drain, spin-up and spin-down currents are generated and flow in opposite directions with nearly equal magnitudes, indicating that the thermal spin current dominates the carrier transport while the thermal electron current is much suppressed. By modulating the temperature, a pure thermal spin current can be achieved. Moreover, a thermoelectric rectifier and a negative differential thermoelectric resistance can be obtained in the thermal electron current. Through the analysis of the spin-dependent transport characteristics, a phase diagram containing various spin caloritronic phenomena is provided. In addition, a thermal magnetoresistance, which can reach infinity, is also obtained. Our results put forward an effective route to obtain a spin caloritronic material which can be applied in future low-power-consumption technology.

Spin caloritronics, i.e., a combination of spintronics and caloritronics in materials, is a research direction that provides alternative strategies for thermoelectric waste heat recovery^1^,^2^ and the future information technologies^3^,^4^,^5^,^6^ A notable recent discovery of spin caloritronics is the observation of spin-dependent Seebeck effect (SDSE)^7^ i.e., a phenomenon that temperature bias can produce a spin current and an associated spin voltage. In order to realize low-power- consumption nanodevices^8^,^9^, we should efficiently utilize spin current while reduce conducting electron current as much as possible, because the electron current usually brings Joule heating^10^,^11^. It is fortunate that the SDSE makes it possible. If the thermally induced spin-up and spin-down currents flow in opposite directions and possess nearly equal magnitudes, a nearly non-dissipative SDSE occurs. For convenience, we call it perfect SDSE. By using the SDSE, Hu *et al.* has achieved spin voltage in CoFeAl nanowire approximately 100 times more than a conventional ferromagnetic material at room temperatures^12^, which provides an effective route to realize high efficient thermal spin injection and generators for driving spintronic devices. The experimental achievements put forward the possibility of applying spin caloritronics in energy harvesting devices to directly convert heat into spin current with low-energy dissipation. However, it is still a huge challenge to realize the perfect SDSE nearly without thermal electron current in a large temperature region.

Graphene, a form of carbon which has a two-dimensional (2D) honeycomb structure^13^, has been explored in spin caloritronics naturally, due to its unique physical properties^14^. Some past works reported that the SDSE can emerge in graphene-based nanodevices^3^. However, the subsequent investigations show that graphene is not an efficient thermoelectric material, since its thermal conductance is extremely low figure of merit^15^,^16^,^17^, which makes it difficult to exhibit the perfect SDSE. It is encouraging that silicene, a monolayer of silicon atoms bonded together on a 2D honeycomb lattice like graphene, has been synthesized successfully very recently^18^,^19^. Compared with graphene, silicene may be more promising in thermoelectrics. First, the electronic structure calculations suggest that silicene is equivalent to graphene^20^, i.e., the electrical conductivity of silicene is as high as that of graphene. Second, silicene is not planar but has a buckled structure, in which the height difference between adjacent Si atoms is about ∆ ≈ 0.46 Å^21^,^22^, leading to a nonzero energy gap and enhancing Seebeck coefficient remarkably^23^,^24^. Besides, silicene is regarded as a new type of atomic-layered material with some outstanding properties^25^,^26^, and can be easily integrated with existing silicon-based electronic devices and technologies. These characteristics make silicene a promising candidate for spin caloritronic materials and stimulate researchers’ wide interest. For example, Yang *et al.* investigated the spin-dependent thermoelectric transport properties of zigzag-edged silicene nanoribbons (ZSiNRs) doped by an Al-P bounded pair at different edge positions, and found that these ZSiNRs can exhibit a temperature-controlled giant thermal magnetoresistance and a high spin-filter efficiency^27^. Nevertheless, a large thermal electron current, which can be compared to the thermal spin current, is still generated in a large temperature region. Considering these, in this work, we devote to designing another type of ZSiNRs with different hydrogen-terminated edges to exhibit the perfect SDSE what we want to achieve.

The ZSiNR heterojunction proposed here is composed of single-hydrogen-terminated ZSiNR (N-ZSiNR-H, sp^2^-hybrid) as the source and double-hydrogen-terminated ZSiNR (N-ZSiNR-H_2_, sp^3^-hybrid) as the drain (see Fig. 1), where N denotes the number of zigzag chains across the ZSiNR. These ZSiNRs could be hopefully fabricated in experiment, since the composition of the sp^2^ and sp^3^-like bonds at the edges might be feasible experimentally by controlling the chemical potential of hydrogen via temperature and pressure of H_2_ gas^28^,^29^. Our theoretical calculations from the nonequilibrium Green’s function (NEGF) method combined with density functional theory (DFT)^30^,^31^,^32^ show that by applying temperature difference between the source and the drain, spin-up and spin-down currents are generated and flow in opposite directions with nearly equal magnitudes. This supports the emergence of the perfect SDSE^5^. Moreover, the thermoelectric rectification (TR), the negative differential thermoelectric resistance (NDTR), and the thermal colossal magneto- resistance effect (TCMR), are also obtained here. Besides, the thermally driven spin currents show an odd-even symmetry. The even-N ZSiNRs are beneficial to exhibit the perfect SDSE, while the odd-N ZSiNRs tend to generate highly spin-polarized currents.

## Results

**Figure 1:**
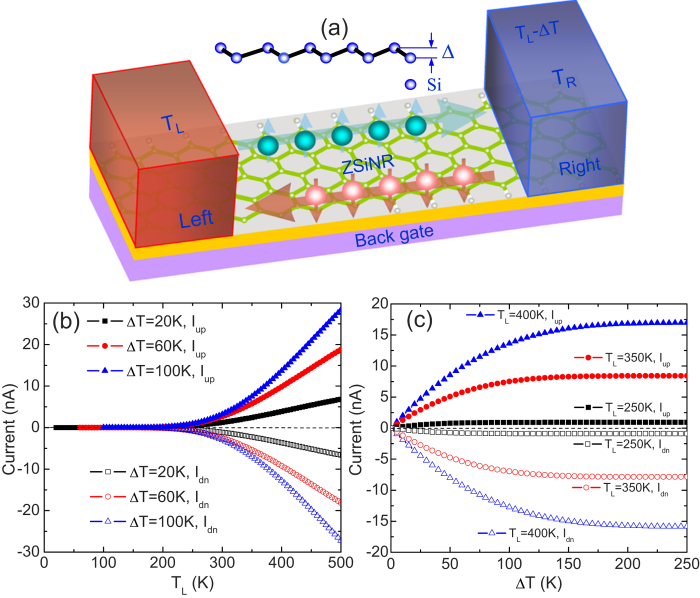
Schematic of the ZSiNR heterojunction and thermal-driven spin currents. (a) The schematic illustration of a (N-ZSiNR-H)/(N-ZSiNR-H_2_) heterojunction, which is spin-polarized and placed on a substrate. ∆T represents the temperature difference between the source (T_L_) and the drain (T_R_), i.e., T_L_–T_R_. (b) Spin currents as a function of T_L_ for different ∆T. The spin-up current I_up_ and the spin-down current I_dn_ flow in opposite directions (spin Seebeck effect). (c) I_up_ and I_dn_ as a function of ∆T for different source temperature T_L_.

First, a schematic illustration of the proposed N-ZSiNR-H and N-ZSiNR-H_2_ heterojunction is shown in Fig. 1(a). Here we focus on the currents driven by temperature difference (∆T) without any external bias voltage, the difference between the temperature of the source (T_L_) and the drain (T_R_), that is, ∆T = T_L_ – T_R_. Simultaneously, we set the back gate and bias voltages as zero, so that carrier concentration and the currents are only determined by temperatures. Except in special notes, the width of the ZSiNR in our discussion is set as 1.2 nm, that is, 4-ZSiNR with four silicon dimmers.

Figure 1(b) describes the spin currents of the (4-ZSiNR-H)/(4-ZSiNR-H_2_) heterojunction versus T_L_ with different ∆T. It is clearly seen that spin currents are generated without any transverse electric field or gate voltage, wherein the spin-up current I_up_ is positive while the spin-down one I_dn_ is negative. Since the spin-up and spin-down currents flow in opposite directions with nearly equal magnitudes, and are generated only by a temperature gradient, thus it is believed that a perfect SDSE emerges in the these ZSiNRs^7^,^10^,^33^. From the curves of spin currents, we find that there is a threshold temperature T_th_ (~250 K). When T_L_ < T_th_, both spin-up and spin-down currents are approximately zero. When T_L_ > T_th_, the spin currents increase linearly with increasing T_L_. Moreover, as ∆T increases, I_up_ and I_dn_ flow just in opposite directions, as shown in Fig. 1(c). It is noted that at a low temperature difference, the spin currents increase linearly, and meanwhile the spin-up and spin-down currents are symmetrical with each other in the whole region of ∆T about the zero-current axis. These characteristics also strongly support the emergence of the perfect SDSE.

**Figure 2:**
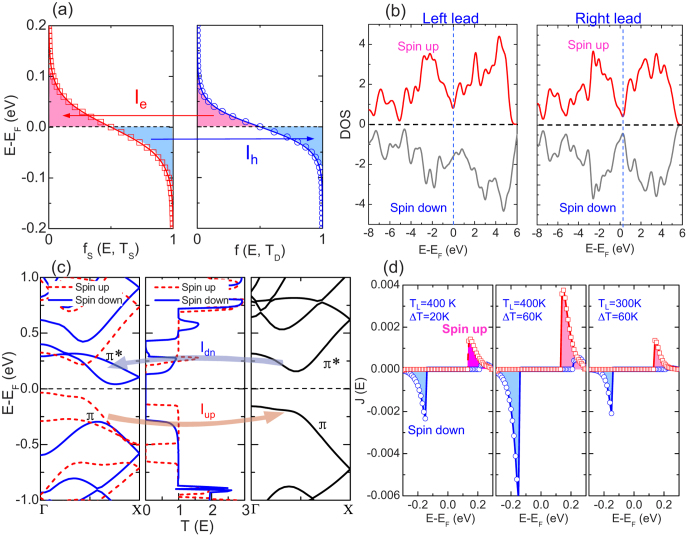
Density of states, band structures and transmission spectra. **** (**a**) The Fermi distribution of the source (the left panel-higher temperature) and the drain (the right panel-lower temperature). The electron current (I_e_) and the hole current (I_h_) are created due to the difference of carrier concentration at the two terminals. (**b**) The density of the states (DOS) of the left panel 4-ZSiNR-H and the right panel 4-ZSiNR-H_2_. (**c**) Band structures of the left panel 4-ZSiNR-H and the right panel 4-ZSiNR-H_2_, spin-dependent transmission spectra (middle panel). The red arrow (blue arrow) illustrates the flowing direction of the spin-up (spin-down) current, respectively, where the transmission is united by e^2^/h. (**d**) The spin-dependent current spectra for different T_L_ and ∆T.

To understand the fundamental mechanism to generate the SDSE in ZSiNRs, we first take into account the electron distribution in the source and the drain, which differs in the carrier concentration and is determined by the Fermi distribution. Since the contacts are the same material and have the similar density of states (DOS), the difference in carrier concentrations between the source and the drain is determined by the Fermi distribution (f_L_(E,T_L_) – f_R_(E,T_R_)), which is intimately related to the electron temperatures at the two terminals as shown in Fig. 2(a). It is clearly demonstrated that carriers (electron) with energy higher than the Fermi energy flow from the source (higher temperature) to the drain (lower temperature), giving rise to electron current I_e_, because the electron distribution of the source is higher than that of the drain. Conversely, carriers (hole) with energy lower than the Fermi energy flow in the opposite directions, resulting in hole current I_h_. If the transmission spectra are symmetric, I_e_ and I_h_ will cancel out each other, leading to zero net thermal current.

To put a further insight into the thermal-induced spin currents and clarify the transport of the carriers, it is necessary to analyze the DOS, the band structures and the transmission spectra of the ZSiNRs. Firstly, the DOS of the system, as expected, shows that the left panel composed of 4-ZSiNR-H has a ferromagnetic ground state, while the right panel composed of 4-ZSiNR-H_2_ has an antiferromagnetic one (see Fig. 2(b)). These results are well consistent with their previous magnetism study by *ab initio* calculations^34^. From the band structures shown in Fig. 2(c), one can find that only the bonding π bond and anti-bonding π^*^ bond appear near the Fermi level, and they are spin-splitting in the source while spin degenerate in the drain. Moreover, as shown in the central panel of Fig. 2(c), near the Fermi level, the spin-dependent transmission spectrum for the heterojunction has a conductance peak in the energy range −0.5 eV < E–E_F_ < −0.12 eV and 0.12 eV < E–E_F_ < 0.28 eV for the spin-up and the spin-down electrons, respectively. These transmission peaks break the electron-hole symmetry in the transmission, leading to the nonzero net spin currents^35^,^36^,^37^. To further illustrate this point, the transmission peak of spin-down electron locates above the Fermi level, and hence, carriers (electrons) can transport from the drain to the source, leading to a negative spin-down current. Conversely, the spin-up electrons show transmission peak below the Fermi level, and the transport of carriers (holes) cause a positive spin-up current from the source to the drain. As a result, the SDSE emerges in the ZSiNR heterojunction. Since the transmission peaks for spin-up and the spin-down electrons are symmetrical with each other with respect to the Fermi level, the spin currents have the same threshold temperature. Because of the exponential decaying nature of the Fermi distribution, and the fact the transmission spectra exhibit a relatively large energy gap for both spin-up and spin-down electrons, a relatively high temperature is required to broaden the distribution to overlap with transmission peaks and then turn on the spin currents. Thus a relatively high threshold temperature T_th_ (~250 K) is observed.

Now, let us give the quantitative analysis of the spin-dependent currents. The current spectra J(E) ( = T(E)(f_L_(E,T_L_)-f_R_(E,T_R_))) of spin-up and spin-down electrons for different temperature settings are shown in Fig. 2(d), where the area covered under the curves associated with the axis of J = 0 reveals the magnitude of the spin currents. At the first glance, one can find that for all temperature settings, the spin-up current spectra are much symmetrical to the spin-down ones about the Fermi level with nearly equal areas, confirming further the emergence of the perfect SDSE. Besides, taking the spin-up spectra for example, when T_L_ is fixed (400 K), the peak of the current spectrum at ∆T = 60 K is higher than that at ∆T = 20 K, indicating that the spin currents increase with increasing ∆T. Nevertheless, it is obvious that the area for J at T_L_ = 300 K is smaller than others, resulting in a substantial increasing spin currents with the growth of T_L_.

**Figure 3:**
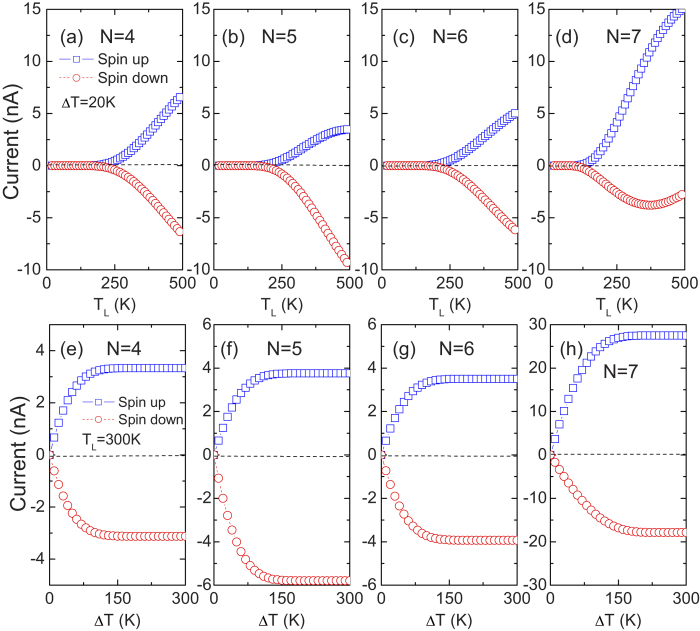
Thermally induced spin currents in different width ZSiNRs. **** (**a**–**d**) The spin currents I_up_ and I_dn_ as a function of the source temperature T_L_ for several heterojunctions (N-ZSiNR-H)/(N-ZSiNR-H_2_) with the temperature difference ∆T = 20K, where the ribbon wider parameter N is adopted from 4 to 7. (**e**–**h**) The spin currents I_up_ and I_dn_ as a function of ∆T for the corresponding ZSiNR heterojunction at T_L_ = 300 K.

Considering the realistic applications of the ZSiNRs, we should explore the influence of the ribbon width on the SDSE. Fig. 3 shows the spin currents I_up_ and I_dn_ versus T_L_ and ∆T for the N-ZSiNR heterojunctions with N = 4 - 7. It is found that as N increases, T_th_ decreases slightly, while the thermally driven spin currents are enhanced, indicating that a large thermal-induced spin current can be obtained in a wider ZSiNR heterojunction. Moreover, the thermal-induced spin currents show a symmetry-dependent characteristic. For the even-N ZSiNRs, I_up_ and I_dn_ are almost symmetrical with each other about the zero-current axis for any values of T_L_ and ∆T. While for the odd-N ZSiNRs, this symmetry is broken. Taking 7-ZSiNR as an example, the values of I_up_ are much larger than these of I_dn_, leading to the polarization of spin current SP ( = (|I_up_| − |I_dn_|)/(|I_up_| + |I_dn_|) × 100) as high as 68.7% at T_L_ = 500 K and ∆T = 20 K, while under the same conditions, SP is just 1.7% in 4-ZSiNRs.

The above spin transport characteristics show that the symmetry still plays an important role on the spin caloritronics of ZSiNRs^36^,^37^. In fact, the even-N ZSiNRs and the odd-N ones have different space groups, and they are *P*2/*m* and *P*21/*m*, respectively. And meanwhile, the characters of the bonding π band and the anti-bonding π^*^ band around the Fermi level (see Fig. 2(c)) determine the transport properties of ZSiNRs. Besides, the even-N ZSiNRs have *c*_2_ symmetry with respect to the central axis parallel to the transport direction. That is to say, after rotating 180° around this axis, namely, *c*_2_ operation (it can also be viewed as an inversion operation with respect to the center axis), the appearance of the even-N ZSiNRs is not changed. Meanwhile, the π and π^*^ wavefunctions of the even-N ZSiNRs have definite parity under the *c*_2_ operation. These characteristics ensure the symmetry of the spin currents flowing in an even-N ZSiNR heterojunction. The odd-N ZSiNRs, however, have no *c*_2_ symmetry with respect to the center axis, so its π and π^*^ bands have no definite parity under *c*_2_ operation. Just due to no restriction of parity, asymmetric thermal spin currents will be generated in the odd-N ZSiNRs. Thus it is believed that the ZSiNRs with different widths tend to exhibit different spin caloritronic behaviors. The even-N ZSiNR heterojunctions are beneficial to exhibit perfect SDSE, while the odd-N ZSiNRs tend to generate high-polarized spin currents.

**Figure 4:**
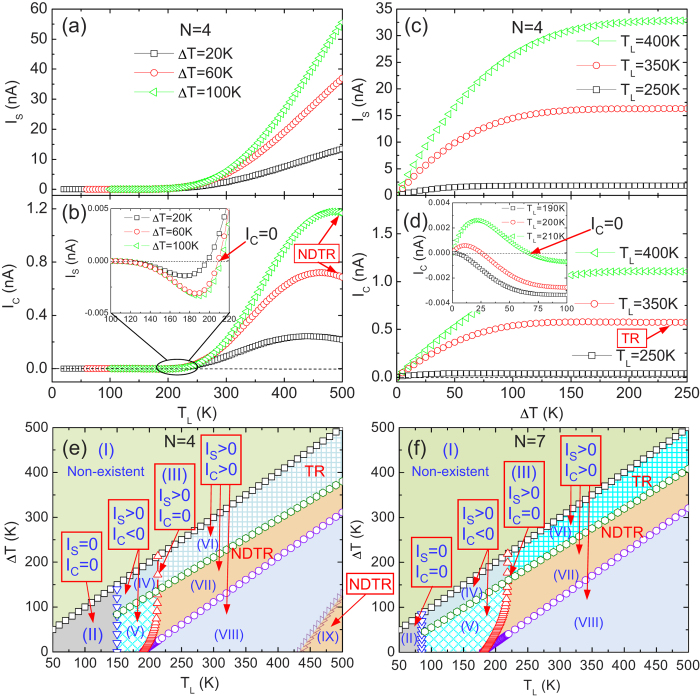
Phase diagrams for total spin and net electron currents. **** (**a**–**b**) The total spin current I_S_ ( = I_up_ − I_dn_) and the net electron current I_C_ ( = I_up_ + I_dn_) in the heterojunction (4-ZSiNR-H)/ (4-ZSiNR-H_2_) versus T_L_ for different ∆T. (**c**–**d**) I_S_ and I_C_ versus ∆T for different T_L_ in the same heterojunction. (**e**) Phase diagram in the (∆T, T_L_) plane for I_S_ and I_C_ flowing through the heterojunction (4-ZSiNR-H)/(4-ZSiNR-H_2_), where nine different kinds of thermoelectric transport regimes, i.e., (I) non-existent region, (II) I_S_ and I_C_ are closed, (III) the thermal-induced pure spin current emerges, (IV) and (VI), thermoelectric rectification (TR) region, in (IV), I_C_ < 0, while in (VI), I_C_ > 0, (V) I_S_ and I_C_ flow in opposite directions, (VII) and (IX), I_S_ and I_C_ flow in the same direction with a NDRT effect in I_C_ and (VIII), I_S_ and I_C_ flow in the same direction. (f) Phase diagram of the thermoelectric transport for another heterojunction (7-ZSiNR-H)/(7-ZSiNR-H_2_).

Since the main aim in this work is to explore the perfect SDSE in the ZSiNR heterojunction, next we investigate the thermal-driven total spin currents I_S_ ( = I_up_ − I_dn_) and the net electronic currents I_C_ ( = I_up_ + I_dn_). Fig 4a–d show I_S_ and I_C_ as a function of T_L_ and ∆T. As predicted, I_S_ increases with increasing T_L_ or ∆T, and its values are nearly two orders of magnitude larger than those of I_C_ in the total-temperature region. This supports that the carrier transport through the heterojunction is dominated by the spin current. Furthermore, as T_L_ or ∆T increases, I_C_ displays as novel transport properties. Taking the case of ∆T = 20 K as an example (see Fig. 4(b)), I_C_ keeps zero as T_L_ is below T_th_. As T_L_ increases, I_C_ increases to its maximum value and then decreases to zero, which indicates that the negative differential thermal resistance (NDTR) occurs. The emergence of the NDRT is a consequence of the competition between I_up_ and I_dn_ with opposite flowing directions. Moreover, as T_L_ increases to a critical temperature T_L1_ = 195K, I_C_ decreases to zero, indicating the appearance of the thermal-induced pure spin current. As T_L_ is beyond T_L1_, I_C_ changes its flowing direction, since its sign is reverse. As T_L_ increases over another critical temperature T_L2_ = 460 K, the NDTR appears again. In addition, as ∆T increases to some larger values (see Fig. 4(d)), the thermoelectric rectification (TR) emerges.

Based on the above characteristics of the spin and charge transports, we can obtain a complete phase diagram in T_L_-∆T plane to show various spin caloritronic phenomena in the 4-ZSiNR heterojunction as shown in Fig. 4(e), which summarizes the main results of this work. First, considering that the device temperature relations T_R_ > 0 and ∆T = T_L_ – T_R_, 0 < ∆T < T_L_ is required, namely, the regime (I) where T_R_ < 0 is non-existent. In the regime (II), I_S_ and I_C_ are both closed due to the energy gap in the bandstructures. In the regime (III), I_S_ > 0 while I_C_ = 0, the thermal-induced pure spin current emerges. In the regimes (IV) and (V), I_S_ > 0 and I_C_ < 0, i.e., I_S_ and I_C_ flow in opposite directions. In the regimes (VI), (VII), (VIII) and (IX), I_S_ > 0 and I_C_ > 0, i.e., I_S_ and I_C_ flow in the same direction. It should be noted that in the regimes (IV) and (VI), TR appears, and in the regimes (VII) and (IX), the NDTR occurs. This phase diagram will help us understand the different thermal-induced transport properties of the ZSiNR heterojunction. For a comparable study, the phase diagram of the spin and charge transports in the 7-ZSiNR heterojunction is drawn in Fig. 4(f). It is found that compared to that of the 4-ZSiNR heterojunction, the phase diagram shows similar characteristics, nevertheless, the regime (III), where the thermal-induced pure spin current emerges, and the boundary between the regime (VI) and (VII), both shifts toward low temperatures. As a result, the area of the NDTR regime becomes larger in a wider ZSiNR heterojunction. Moreover, the regime (IX), where the NDTR occurs in the 4-ZSiNR heterojunction, while disappears from a wider ZSiNR one.

**Figure 5:**
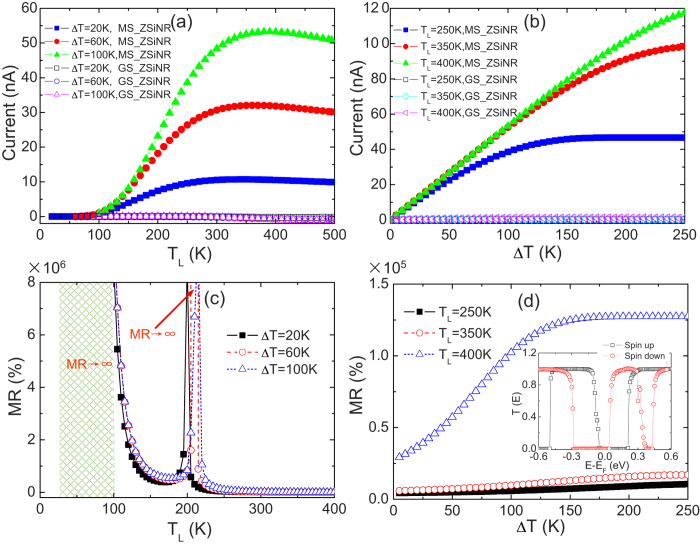
Thermal colossal magnetoresistance in the 4-ZSiNR heterojunction. **** (**a**) The electron currents as a function of T_L_ for the GS-ZSiNR and the M-ZSiNR with ∆T = 20, 60 and 100 K. (**b**) The net electron currents as a function of ∆T for the GS-ZSiNR and the M-ZSiNR with T_L_ = 250, 350 and 400 K. (**c**) The thermal magnetoresistance (MR) as a function of T_L_ with different ∆T by translating ZSiNRs from ground state to ferromagnetic state. MR is calculated based on the following formula: MR = (R_GS_–R_M_)/R_M_ × 100 = ((|I_M_|)/(|I_GS_|)–1) × 100, where R_GS_ = ∆T/|I_GS_| and R_M_ = ∆T/|I_M_| are the thermal-induced resistances in the GS-ZSiNR and the M-ZSiNR, respectively. (**d**) MR as a function of ∆T with different T_L_ in the same heterojunction, and the inset shows the transmission spectrum of the M-ZSiNR heterojunction by applying an external magnetic field.

Finally, we investigate the thermal magnetoresistance (MR) of the ZSiNR heterojunction changing from ground state (GS, without any external magnetic field) to the magnetic state (MS, an external magnetic field is applied to the heterojunction), which can be obtained from the equation MR (%) = (R_MS_ – R_GS_)/(R_GS_) × 100 = [(I_GS_ – I_MS_)/I_MS_] × 100, where I_GS_ and I_MS_ are the total electronic currents in the GS and MS state^38^. As shown in Fig. 5(a,b), the I_GS_ of the GS-ZSiNR nearly equals to zero, because I_up_ and I_dn_ flow in opposite directions with nearly equal magnitudes. However, the I_MS_ in the MS-ZSiNR is much larger under the same conditions, and increase rapidly even linearly as T_L_ or ∆T increases. These transport behaviors can be understood from its spin dependent transmission spectrum of the ferromagnetic state as shown in the set of Fig. 5(c). We found that the energy gap in the spin-dependent transmission spectrum of the MS-ZSiNR is much smaller than that of the GS-ZSiNR. Therefore, the presence of the external magnetic field in ZSiNRs can change the thermal-induced currents, that is, varying MR of the system remarkably. Because there is no any electric current in the regimes (II) and (III) of the phase diagram in Fig 4(e,f), the MR ratio can reach infinity in these two particular regimes as the GS-ZSiNR changes to the MS-ZSiNR (see Fig. 5(c)). Obviously, the thermal colossal magnetoresistance (CMR) of ZSiNRs can be achieved at room temperatures and surpasses the conventional metal-based MR devices^39^.

## Discussion

In summary, we have proposed a new configuration of hydrogen-terminated ZSiNR heterojunction to realize the SDSE. By breaking the electron-hole symmetry, spin-dependent currents can be generated in ZSiNRs by using temperature difference instead of external electric bias. The spin-up and spin-down currents flow in opposite directions with nearly equal magnitudes, thus the conducting electron current in the heterojunction is much reduced and the related Joule heating is much suppressed, indicating its potential applications in future low-power-consumption technology. It is noted that this thermal-induced spin current can be detected in experiment. First, considering that only two edges of ZSiNRs are hydrogen-modified, the conductance channels for spin-up and spin-down electrons are mainly located in the two edges of the nanoribbons^3^. The completely spin-polarized currents in both edges can be detected by the degree of circular polarization state of the emitted light from (Al, Ga) As diode^40^,^41^. Then, as similar as other types of spin currents, the thermal-induced spin currents can be transformed into the current by the inverse spin Hall effect^42^,^43^, thus even a very small thermal-induced spin current in our ZSiNRs can still be detected by electric method in experiment.

Apart from the above, the thermally induced spin currents in ZSiNRs show an odd-even symmetry, the even-N ZSiNRs are beneficial to exhibit the SDSE nearly without net electron current, while the odd-N ZSiNRs tend to generate high-polarized spin currents. Based on the analysis of the spin and charge transports in ZSiNRs, we obtain a complete phase diagram describing various spin caloritronic phenomena. In addition, an external magnetic field can completely switches on the net electron current in the heterojunction, strongly supporting that the thermal-induced CMR effect is obtained in our device, and the CMR value can reach infinity. The results in this study put forward an effective route to realize the SDSE nearly without conducting electron current in silicene-based spin caloritronic materials.

## Methods

Our calculations have been performed with density functional theory (DFT) combined with nonequilibrium Green’s function technique (NEGF). First, geometry optimization and electronic structure calculations are performed with the double numerical plus polarization (DNP) basis set implemented in the SIESTA code^44^. The positions of the atoms are relaxed until the maximum force on each atom is no more than 0.01eV/Å. Then, we calculated the transmittances using the TRANSAMPA 2 code^45^,^46^. The core electrons are described by norm-conserving pseudopotentials, and the local spin density approximation (LSDA) with the Perdew-Burke-Ernzerhof generalized gradient approximation^47^ for the exchange-correlation functional. A double-zeta-polarized (DZP) basis set is used and the cut off energy is 150Ry and a Monkhorst-Pack *k*-mesh of 1 × 1 × 100 is chosen in our work. In addition, the optimization result shows that Si-Si distance is 2.27Å, larger than C-C length. In the Landauer-Büttiker formalism, the spin-dependent current through the system is given by^48^
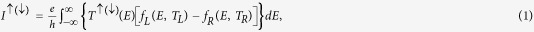
where *e* is the electron charge, *h* is the plank constant, 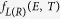 is the equilibrium Fermi-Dirac distribution for the left (right) electrode, *T*_*L*(*R*)_ is the temperature of the left (right) contract, and 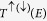 is the spin resolved transmittance function and can be defined as
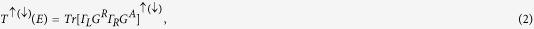
where *G*^*R*(*A*)^ is the retarded (advanced) Green’s functions of the central region and Γ_*L*(*R*)_ is the coupling matrix of the left (right) contact. These expressions will help us obtain the thermal spin-dependent transports through the ZSiNR heterojunction.

## Author Contributions

H.F. supervised the whole work, analyzed the results, carried out partial numerical calculations and wrote the paper. D.W. and Z.Z. built the device structure and carried out the numerical calculations. D.W., Z.Z. and L.G. made a discussion and analyzed the results. All authors reviewed the manuscript.

## Additional Information

**How to cite this article**: Fu, H.-H. *et al*. Spin-dependent Seebeck Effect, Thermal Colossal Magnetoresistance and Negative Differential Thermoelectric Resistance in Zigzag Silicene Nanoribbon Heterojunciton. *Sci. Rep.* **5**, 10547; doi: 10.1038/srep10547 (2015).
